# Age differences in spontaneous cerebrovascular reactivity at rest

**DOI:** 10.1007/s11357-025-01935-9

**Published:** 2025-10-30

**Authors:** Allison C. Engstrom, Shubir Dutt, John Paul M. Alitin, Arunima Kapoor, Trevor Lohman, Isabel J. Sible, Anisa J. Marshall, Fatemah Shenasa, Jillian L. Joyce, Aimée Gaubert, Farrah Ferrer, Amy Nguyen, Xingfeng Shao, Danny J. J. Wang, Daniel A. Nation

**Affiliations:** 1 Department of Psychological Science, Social and Behavioral Sciences Gateway, University of California, Irvine, 4201, Irvine, CA 92697-7085 USA; 2 Memory and Aging Center, Department of Neurology, Weill Institute for Neurosciences, University of California, San Francisco, 1651 4Th St Suite 212, San Francisco, CA 94158 USA; 3 Leonard Davis School of Gerontology, University of Southern California, 3715 McClintock Ave, Los Angeles, CA 90089 USA; 4 Department of Psychology, G. Mudd Building, University of Southern California, 3620 McClintock Ave, Los Angeles, CA 90089 USA; 5 Stevens Neuroimaging and Informatics Institute, University of Southern California, 2025 Zonal Ave, Los Angeles, CA 90033 USA; 6 Keck School of Medicine, University of Southern California, 1975 Zonal Ave., Los Angeles, CA 90033 USA

**Keywords:** Cerebrovascular reactivity, Cerebral blood flow, Aging, Older adults

## Abstract

Deficits in cerebral blood flow (CBF) and cerebrovascular reactivity (CVR) may indicate vulnerability to vascular dysfunction and cognitive impairment. Prior findings on age-related change in CVR are mixed, and no studies to date have investigated age group differences in spontaneous CVR (sCVR) at rest. The present study compared whole brain and regional sCVR at rest between 24 healthy younger adults (ages 18–34) and 163 cognitively unimpaired older adults (ages 55–87). Relative to younger adults, older adults had deficits in both whole brain and regional sCVR at rest. Among the regions examined, medial temporal lobe areas showed the largest age differences in sCVR at rest. Age differences in sCVR and CBF variability were larger than age differences in mean CBF. Results show age-related deficits in sCVR are present even in cognitively unimpaired older adults, suggesting sCVR may be indicative of cerebrovascular aging. Further research should investigate sCVR as a means for early detection of age-related vascular dysfunction that may predate irreversible vascular brain injury.

**Supplementary Information:**

The online version contains supplementary material available at https://doi.org/10.1007/s11357-025-01935-9.

## Introduction

Cerebrovascular health is crucial for maintaining cognitive function across the lifespan, as vascular dysfunction has been linked to age-related cognitive decline, neurodegeneration, and dementia [1–7]. In addition to large vessel disease, cerebral microvascular dysfunction characterized by deficits in blood–brain barrier permeability, cerebral perfusion, and cerebrovascular reactivity (CVR) may play a key role in cognitive impairment [2, 3, 8–10]. CVR reflects the ability of cerebral vessels to regulate blood flow in response to homeostatic or experimentally induced CO₂ changes [2, 11, 12]. The majority studies have focused on CVR responses to experimentally manipulated CO_2_ levels through methods such as gas inhalation or breathing paradigms. However, fluctuations in cerebral blood flow (CBF) also occur in response to spontaneous changes in end-tidal CO₂ levels at rest. While spontaneous blood pressure fluctuations can also influence CBF through autoregulatory mechanisms, prior work has demonstrated that, under resting and normotensive conditions, spontaneous BOLD/CBF fluctuations are more strongly coupled to end-tidal CO₂ variation than to blood pressure [13, 14]. Thus, spontaneous CVR (sCVR) responses are generally interpreted as reflecting CO₂-driven fluctuations, which can be measured using perfusion or blood oxygen level–dependent (BOLD) signals combined with capnographic monitoring. Studies to date suggest that resting state methods may provide similar results to those of experimentally induced CVR [15]. For example, recent work from our group indicates both experimentally induced CVR responses to hypercapnia and sCVR at rest are deficient in medial temporal regions in older adults with memory deficits relative to those who are cognitively unimpaired [8, 10]. Further study of sCVR is warranted since the relative ease of acquisition may provide an opportunity to assess cerebrovascular function in clinical settings where experimentally induced CVR is difficult or impossible [15].

Despite these convergent findings, other studies suggest sCVR and experimentally induced CVR may be physiologically distinct. For example, a study comparing dynamic changes in sCVR at rest to those of induced hypercapnia using BOLD fMRI found considerable difference in the response of the cerebrovasculature to smaller spontaneous fluctuations in CO_2_ compared to larger, induced changes [16]. CVR responses to experimentally induced changes in CO_2_ levels may quantify vascular reserve, endothelial function, and impairment in vasodilation, and sCVR responses at rest may additionally reflect changes to natural variability in cerebral autoregulation and autonomic control [16–18]. Existing studies on sCVR have almost exclusively utilized resting-state BOLD fMRI to obtain and compute CVR values. Perfusion imaging with pseudocontinuous arterial spin-labeling (pCASL)-MRI has been less studied and offers the advantage of more quantitative and direct measurement of tissue perfusion [12, 19]. The present study’s aim is to measure sCVR using pCASL MRI.

In an initial study of sCVR in older adults using pCASL-MRI, we reported an association between medial temporal lobe sCVR deficits and memory impairment [8]. However, the effects of aging itself on sCVR at rest remain largely unexplored. Findings from experimentally induced CVR are mixed, with some suggesting that deficits in CVR responses can be detected even in cognitively unimpaired older adults[9, 20–25], while other studies using transcranial doppler ultrasound have found increased large vessel CVR responses in older adults [19, 26]. No studies to date have investigated differences in sCVR between younger and older adults using pCASL quantification of CBF. The present study seeks to fill this gap by comparing whole brain and regional pCASL-MRI sCVR at rest between healthy younger adults and cognitively unimpaired older adults. Based on prior studies comparing CVR responses to experimentally induced hypercapnia, we hypothesize that that both global and regional sCVR will be attenuated in cognitively unimpaired older adults compared healthy younger adults.

## Materials and methods

### Participants

A total of 170 older adults (age 55–89) and 28 young adults (age 19–34) were recruited from the Los Angeles County and Orange County communities via outreach events, mailing lists, word-of-mouth, online portals, and other modes of community outreach facilitated by the University of Southern California (USC) Leonard Davis School of Gerontology, and the University of California Irvine (UCI) Alzheimer’s Disease Research Center. All procedures were conducted as a part of the Vascular Senescence and Cognition (VaSC) Study at USC and UCI. All participants were independently living at the time of recruitment. Study exclusion criteria were a prior diagnosis of dementia, history of clinical stroke, family history of dominantly inherited neurodegenerative disorders, current neurological or major psychiatric disorders that may impact cognitive function, history of moderate-to-severe traumatic brain injury, current use of medications that may impair the central nervous system (i.e., anticholinergics, benzodiazepines, barbiturates, etc.), pulmonary conditions that may impact gas exchange, current organ failure or other uncontrolled systemic illness, and contraindications for brain MRI. Eligibility for the study was verified via clinical interview and review of current medications with both the participant, and an informant study partner when available. Additionally, after MRI acquisition, each participant’s T2-weighted scan was screened by a board-certified neuroradiologist for any incidental findings, including silent infarctions. None of the participants in the sample had evidence of any silent infarctions or other exclusionary abnormalities.

### Neuropsychological screening

Older adult participants underwent comprehensive neuropsychological assessment by a trained technician or doctoral student under the supervision of a licensed clinical neuropsychologist in order to screen for cognitive impairment, which included tests of global cognition, memory, attention/executive function, and language. Test findings were reviewed by a blinded neuropsychologist (DAN) and those meeting Bondi/Jak neuropsychological criteria for cognitive impairment were identified [27]. We previously reported deficits in sCVR at rest in older adults with cognitive impairment versus those who were cognitively unimpaired[8]. Thus, older adult participants that met criteria for cognitive impairment (*n* = 34) were excluded from the present study.

### Neuroimaging and cerebral perfusion

All participants completed a brain MRI on a 3 T Siemens MAGNETOM Prisma System scanner. At both sites, the direction of acquisition was anterior to posterior. The present study analyzed the following sequences: 3D T1-weighted anatomical scan for qualitative assessment of brain structures and abnormalities (scan parameters: TR = 2300 ms; TE = 2.98 ms; TI = 900 ms; flip angle = 9 deg; FOV = 256 mm; resolution = 1.0 × 1.0 × 1.2 mm^3^; scan time = 9 min), and 3D gradient and spin-echo (GRASE) pCASL for CBF quantification. The scan parameters were as follows for pCASL: TR = 5000 ms; TE at University of Southern California = 36.3 ms; TE at University of California, Irvine = 37.46 ms; FOV = 240 mm; resolution = 2.5 × 2.5 × 3.4 mm^3^; slice thickness = 3.42 mm; number of slices = 24; labeling duration = 1517 ms; post-labeling delay = 2000 ms. There was a total of 32 acquisitions (1 M0 image + 1 dummy image + 30 alternating tag and control images), with a total scan time of 5 min 25 s, resulting in 15 tag-control pair images.

The pCASL images were pre-processed using the previously reported method in ASLtbx pipeline, applied in SPM12 within MATLAB[28, 29]. Pre-processing steps for pCASL scans included motion correction, co-registration to individual participants’ structural T1-weighted image, spatial smoothing with a 6 mm full-width at half-maximum Gaussian kernel, masked to remove the background, and tag-control subtraction resulting in 15 tag-control pairs for each participant with values for absolute CBF (mL/100 g tissue/min). All CBF images were thresholded below 10 or above 150 mL/100 g/min to exclude CBF outside the anticipated physiological range of gray matter [30, 31]. These CBF quantification maps are generated in T1 space. Then tag-control pairs were warped to 2 mm MNI space and averaged to create mean CBF maps in for each participant. Resulting mean CBF maps were visually inspected for quality and gross abnormalities (i.e., large signal dropout). Partial volume correction was performed by applying participant-specific gray matter masks derived from the gray matter tissue class segmentation of T1-weighted structural images[32]. Segmented gray matter maps were thresholded at 0.3[33], binarized, warped into 2 mm MNI space, and multiplied by the mean CBF maps so that CBF was restricted to gray matter.

Capnography indexed _ET_CO_2_ during MRI acquisition using an M3015A sidestream CO_2_ extension module (Philips Medical Systems) connected to a nasal cannula into which participants breathed. To correct for sampling tubing latency, _ET_CO_2_ time series were shifted by a pre-calibrated duration of time (i.e., 10 s for the present study). This time shift was consistent across sites. For the baseline pCASL scan, _ET_CO_2_ was extracted from the raw time series in accordance with the breathing rate (i.e., at every expiration). Participants were provided with both verbal and visual instructions to “keep your eyes open and stay awake. Breathe as you normally do. Breathe only through your nose. Stay as still as possible”. Participants were excluded from analysis if they failed to adhere to breathing instructions (e.g., lack of positive peaks in raw data suggesting that participant might be breathing through the mouth) or if values fell outside expected physiological range (negative values).

### CVR maps

Spontaneous CVR has been quantified variably in the literature [17]. For consistency with prior studies from our group and others, CVR was conceptualized as the percent change in CBF per unit change in _ET_CO_2_ [8, 12, 34]. Specific computational approach has been previously outlined [23, 35]. In the present study, participant’s individual CVR maps were generated using resting state CBF with simultaneous capnography with the following Eq. (1) computed in each voxel:

1 $$CVR (\%\Delta CBF change/\Delta mmHg)=\frac{100 X ({CBF}_{maximum}-{CBF}_{minimum})/{CBF}_{minimum}}{{ETCO}_{2maximum}-{ETCO}_{2minimum}}$$

**Fig. 1:**
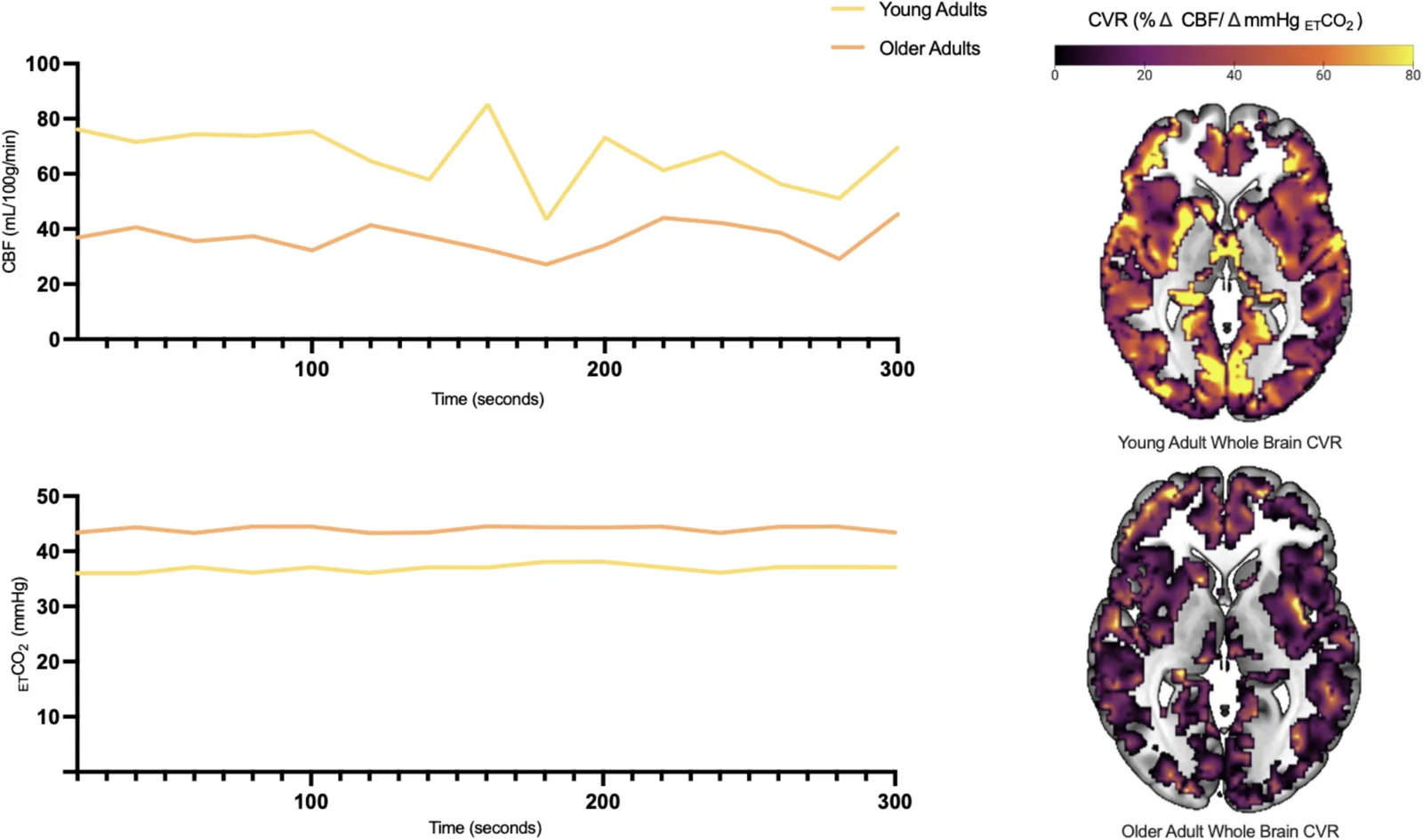
sCVR mapping methodsCVR is calculated by taking percent change in CBF from resting state perfusion values across scan time and divided by unit change in _ET_CO_2_ on capnography. sCVR maps are created and whole brain and regional values are extracted. Representative sCVR maps are displayed from a young adult (age 25) and an older adult (age 75)

Figure 1 provides a visual overview of this method. Based on prior studies and our hypotheses, whole brain and regional mean CVR values were extracted for our regions of interest [36]. Regional mean CVR maps and values were extracted using the Automated Anatomical labeling Atlas 3 [37]. The medial temporal lobe region was concatenated using Wake Forest University’s Pickatlas [38] and included Brodmann areas 27 (rostral parahippocampal gyrus), 28 (ventral entorhinal cortex), 34 (dorsal entorhinal cortex), and 35 and 36 (perirhinal cortex) (see Table 1 for all regions examined).

**Table 1:** Participant demographics

	Total (*N* = 153)	Older adults (*N* = 129)	Younger adults (*N* = 24)
Age, *M (SD)*	62.6 (18.8)	70.08 (7.75)	22.5 (2.40)
Age range	18–89	54–89	18–34
Sex, female, *n* (%)	108 (70.59%)	88 (68.22%)	20 (83.33%)
Dementia Rating Scale raw score, *M (SD)*	141.20 (3.07)	140.30 (3.03)	144.00 (0.00)
^1^Handedness, *n* (%)			
Right	N/A	114 (88.37%)	N/A
Left	N/A	15 (11.63%)	N/A
Vascular risk factors, *n* (%)			
Hypertension	47 (30.72%)	47 (36.43%)	0 (0.00%)
^2^High cholesterol	60 (39.22%)	60 (46.51%)	0 (0.00)
Diabetes	15 (9.80%)	15 (11.63%)	0 (0.00%)
History of smoking	44 (28.76%)	44 (34.11%)	0 (0.00%)
Cardiovascular disease	13 (8.50%)	13 (10.08%)	0 (0.00%)
Atrial fibrillation	6 (3.92%)	6 (4.65%)	0 (0.00%)
Transient ischemic attack	2 (1.31%)	2 (1.55%)	0 (0.00%)
^3^Vascular risk factor total, *M (SD)*	1.13 (1.13)	1.43 (1.09)	0 (0.00%)
^4^Race, *n* (%)			
White	N/A	103 (79.84%)	N/A
Black or African American	N/A	2 (1.55%)	N/A
American Indian or Native Alaskan	N/A	2 (1.55%)	N/A
Native Hawaiian or other Pacific Islander	N/A	1 (0.78%)	N/A
Asian	N/A	19 (14.73%)	N/A
Other	N/A	2 (1.55%)	N/A
^5^Ethnicity, *n* (%)			
Hispanic/Latino	N/A	7 (5.43%)	N/A
Not Hispanic/Latino	N/A	122 (94.57%)	N/A

^1,4,5^Handedness, race, and ethnicity data not available for the younger adult cohort

^2^High cholesterol reflects a self-reported diagnosis of high cholesterol from the participant’s medical doctor

^3^Vascular risk factor total is defined as the sum of participant’s vascular risk factors listed in the above table

### Vascular risk factors and blood pressure

The older adult participants’ vascular risk factor (VRF) burden was evaluated through clinical interviews with the participant and a knowledgeable informant (when available) and included a history of cardiovascular disease (heart failure, angina, stent placement, coronary artery bypass graft, intermittent claudication), hypertension, hyperlipidemia, type 2 diabetes, smoking, atrial fibrillation, and transient ischemic attack or minor stroke. The total VRF burden was defined by the sum of these risk factors and then dichotomized into lower vs. higher burden (0–1 vs. 2 + VRFs) based on prior studies [3, 39, 40]. Additionally, supine blood pressure measurements were collected from the participants prior to the scan using a TeslaDUO MRI Patient Monitor. These data were used for sensitivity analyses.

### Cluster size

For voxel-based analyses presented in this study, Ke refers to the cluster extent, expressed as the number of contiguous voxels surviving the statistical threshold. This metric reflects the spatial extent of significant effects and is reported alongside peak coordinates and statistical values to characterize the robustness and distribution of observed clusters.

### Data availability

The data that support the findings of this study are available upon reasonable request from the corresponding author based on the conditions outlined by the Data Availability Policy and Statement.

## Statistical analysis

**Fig. 2:**
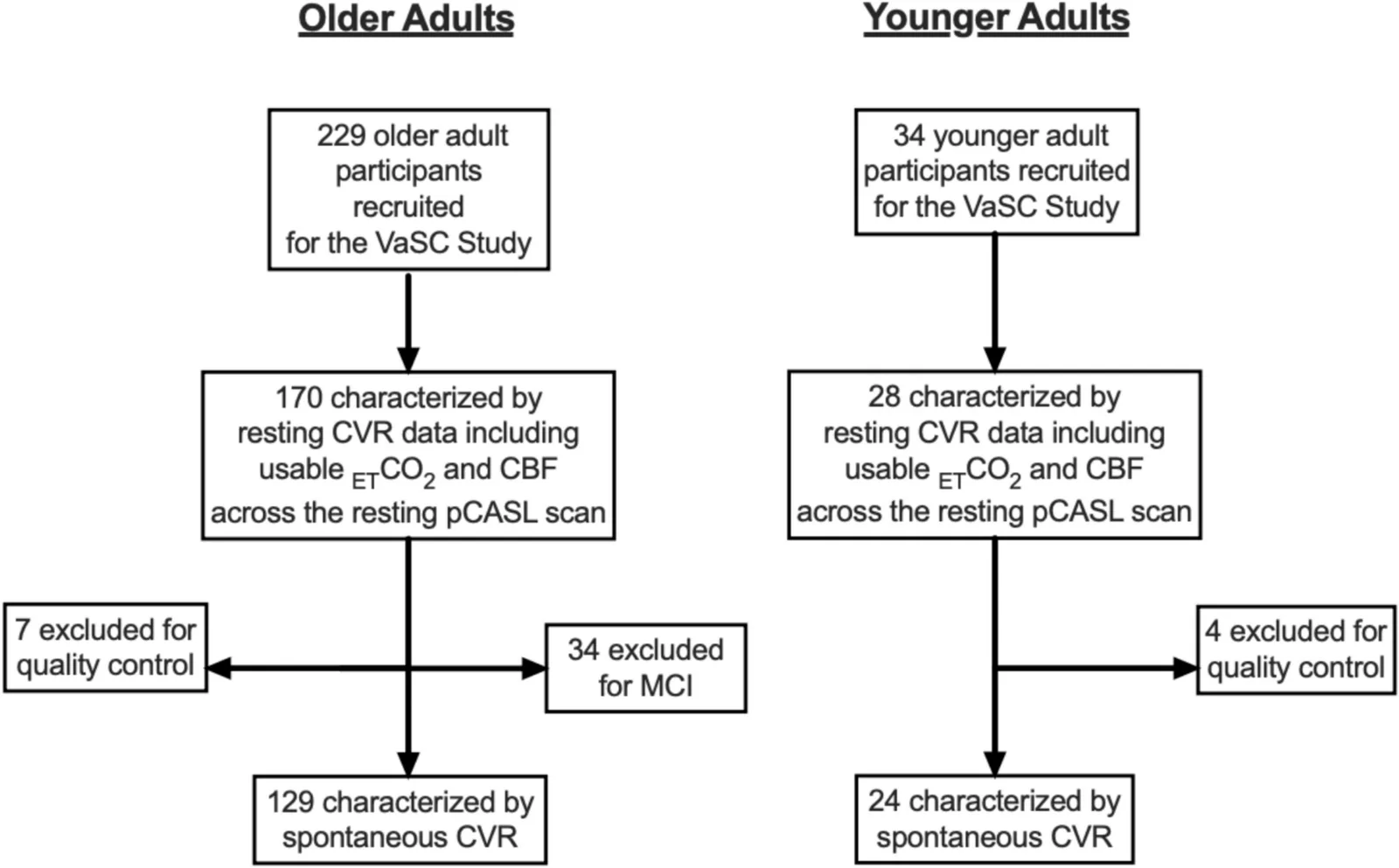
Participant flow diagram

Data was screened for outliers, in which participants were removed if they were greater than +/− 3 SD from the mean. A total of four young adults and seven older adults were excluded during CVR quality control (Fig. 2). Analyses were preformed using SPSS (Version 29.0.1.1) and R (Version 4.3.3). Age comparisons of sCVR for each region-of-interest (ROI) were examined using analysis of covariance (ANCOVA), adjusting for sex. Age comparisons of whole brain CBF and CBF variability were also performed using ANCOVA, adjusting for sex. Additional models controlling for mean CBF were also evaluated. In addition to the ROI analysis, voxel-wise analyses were conducted using SPM12 to provide an unbiased assessment of regional differences in sCVR between older and younger adults. These analyses employed a ANCOVA model with age group as the between-subject factor and sex as a covariate. Statistical maps were thresholded at a voxel-level *p* < 0.001 (uncorrected), with a cluster-level family-wise error (FWE) correction at *p* < 0.001 to control for multiple comparisons.

Sensitivity analyses were conducted using ANCOVA to determine if the results were impacted by high VRF burden in some of the older adult sample. To address this issue, all findings were confirmed by comparison of a smaller sample of older adults (*n* = 87) with low VRF burden (0–1 risk factor) to the younger adult group. To address the mismatch in sample size between older and younger adults, we also confirmed all findings by comparing a sex-matched and sample size-matched group of older adults to the younger adult group. Lastly, to account for the potential influence of blood pressure on sCVR, a sensitivity analysis was conducted in a subset of younger (*n* = 18) and older (*n* = 107) adults in which mean arterial pressure (MAP) was available.

False Discovery Rate (FDR) correction was used to account for multiple comparisons [41].

## Results

After study exclusions, a total of 129 older (mean age = 70.0, *SD* = 7.7, 68.2% female) and 24 younger adults (mean age = 22.5, *SD* = 2.4, 83.3% female) were studied and characterized by demographics (Table 1) and whole brain and regional sCVR data for statistical analysis.

Older adults exhibited significantly diminished whole brain sCVR compared to younger adults (older adults mean = 21.06, *SD* =, 11.08, younger adults mean = 45.83, *SD* = 33.61, *F* = 44.76, *p* = 0.0000000042; 54.05% lower sCVR in older relative to younger adults).

### ROI analysis

Older adults showed significantly lower sCVR than younger adults in all regions studied (Table 2), except the thalamus which showed a nonsignificant trend toward lower levels in older adults relative to younger adults (*p* = 0.06). Among significant age differences in regional sCVR, medial temporal lobe regions showed the largest effect sizes (*η*_*p*_^2^ range = 0.221–0.246; between 55.17% and 60.78% lower sCVR in older relative to younger adults), including the entorhinal cortex and parahippocampal cortex. Age differences remained significant in models controlling for mean CBF, with the parahippocampal gyrus having the largest effect size (η_p_^2^ = 0.149) (see Supplementary Table 1). All ROI analyses were included for FDR correction. All statistically significant findings survived the FDR correction.

**Table 2:** Comparison of mean regional sCVR values for younger and older adults at rest, controlling for sex

Brain region	Younger adults (*N* = 24) *M (SD)*	Older adults (*N* = 129) *M (SD)*	Mean difference *M (SE)*	*F*	*p*	*η*_*p*_^2^
Whole brain	45.83 (33.61)	21.06 (11.08)	24.96 (3.73)	44.76	< 0.001	0.230
Superior frontal gyrus	28.89 (16.93)	20.35 (14.18)	8.55 (3.27)	6.831	0.010	0.051
Middle frontal gyrus	35.45 (31.33)	21.51 (14.63)	14.13 (4.11)	22.92	< 0.001	0.134
Inferior frontal gyrus	53.54 (44.02)	27.27 (17.61)	25.96 (5.01)	22.92	< 0.001	0.134
Medial orbitofrontal cortex	61.45 (49.78)	32.34 (26.75)	30.26 (7.86)	14.83	< 0.001	0.105
Lateral orbitofrontal cortex	57.80 (49.51)	32.19 (24.97)	25.91 (7.09)	13.36	< 0.001	0.088
Anterior cingulate cortex	44.37 (29.59)	27.27 (16.94)	17.85 (4.49)	15.82	< 0.001	0.102
Posterior cingulate cortex	29.40 (17.39)	19.61 (12.95)	12.01 (3.18)	14.30	< 0.001	0.097
Precuneus	36.75 (21.91)	21.12 (13.43)	16.35 (3.42)	22.87	< 0.001	0.134
Inferior parietal cortex	34.79 (27.51)	19.08 (16.39)	15.46 (4.39)	12.42	< 0.001	0.085
Inferior temporal gyrus	35.34 (26.87)	19.57 (13.68)	15.85 (3.99)	15.76	< 0.001	0.107
Medial temporal lobe	47.88 (36.02)	18.78 (14.14)	29.12 (4.52)	41.59	< 0.001	0.234*
Perirhinal cortex	33.94 (22.24)	24.96 (18.45)	9.66 (4.54)	4.53	0.035	0.032
Entorhinal cortex	46.91 (31.93)	21.03 (15.05)	26.70 (4.67)	32.65	< 0.001	0.221*
Parahippocampal gyrus	51.01 (36.78)	20.45 (13.87)	30.33 (4.49)	45.61	< 0.001	0.246*
Hippocampus	42.13 (32.99)	22.41 (15.15)	19.82 (4.50)	19.38	< 0.001	0.125
Amygdala	53.87 (34.77)	29.01 (19.53)	24.87 (5.24)	22.54	< 0.001	0.145*
Caudate	45.14 (38.17)	24.15 (17.55)	31.49 (5.31)	16.39	< 0.001	0.117
Thalamus	30.10 (19.72)	21.84 (17.07)	7.74 (4,13)	3.52	0.063	0.027

*Large effect sizes: *η*_*p*_^2^ > 0.14 [42]

**Fig. 3:**
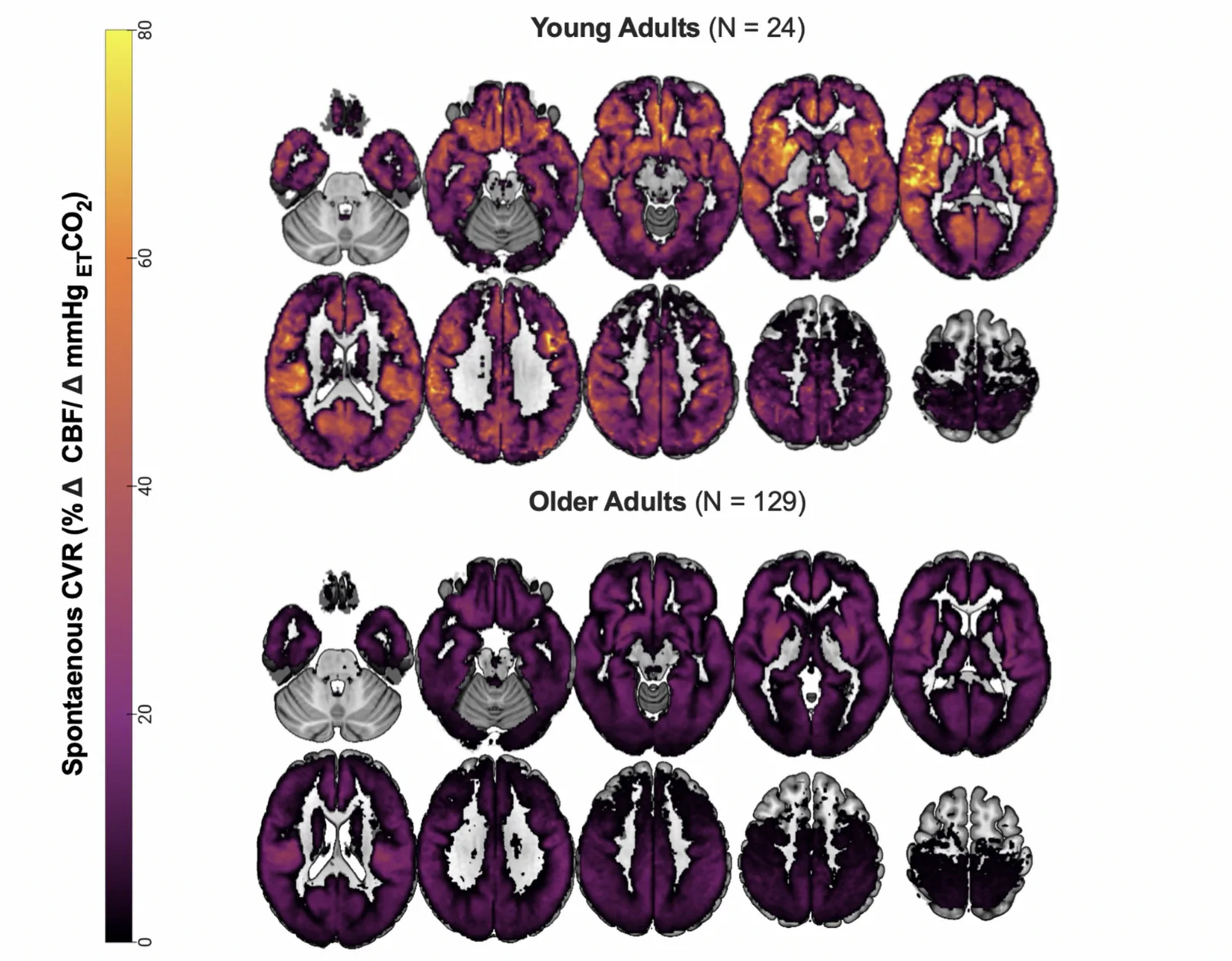
Whole brain grey matter average sCVR maps are displayed for visual comparison between the younger adult and older adult groups

Visual comparison of whole brain sCVR maps of younger and older adults at rest is consistent with these statistical results, indicating attenuated sCVR response in older adults relative to younger adults (Fig. 3).

### CBF and CBF variability

Age differences in whole brain CBF were also investigated and indicated significantly lower resting CBF in older adults compared to younger adults (older adults mean = 49.22, *SD* = 7.47, younger adults mean = 63.70, *SD* = 16.57, *F* = 49.52, *p* = 0.000000000057; 22.73% lower CBF in older relative to younger adults). Older adults also showed significantly lower CBF variability (measured by maximum minus minimum CBF during 5 min of pCASL-MRI) than younger adults (older adults mean = 20.40, *SD* = 7.66, younger adults mean = 36.35, *SD* = 11.74, *F* = 78.37, *p* = 0.0000000000000017; 62.45% lower CBF variability in older relative to younger adults). Results maintained significant when controlling for mean CBF (older adults mean = 20.40, *SD* = 7.66, younger adults mean = 36.35, *SD* = 11.74, *F* = 43.12, *p* = 0.00000000072; 62.45% lower CBF variability in older relative to younger adults).

### Voxel-based morphometry analysis

**Fig. 4:**
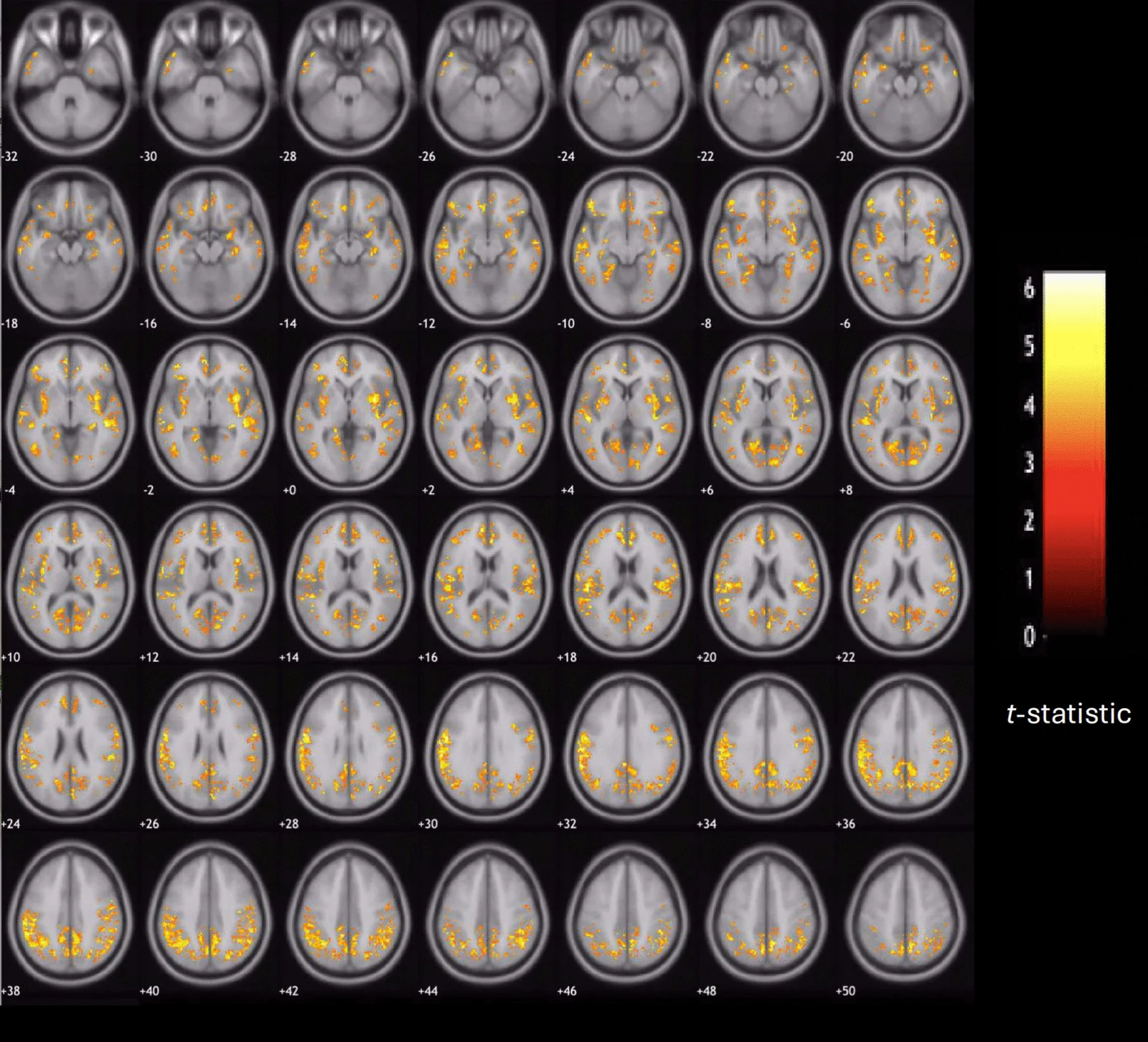
Voxel-based morphometry results

VBM ANCOVA revealed significant deficits in regional sCVR in older adults compared to younger adults in 12 different clusters. These clusters include regions in the left medial frontal cortex, bilateral anterior cingulate gyrus, bilateral anterior insula, bilateral posterior insula, bilateral parietal and central operculum, bilateral precuneus, right calcarine cortex, right cuneus, right inferior and middle occipital gyrus, bilateral transverse temporal gyrus, left superior temporal gyrus, bilateral planum polare, right hippocampus, right putamen, and right thalamus. These relationships maintained significant after family-wise error correction at *p* = 0.001. VBM results are shown in Fig.4 and Table 3.

**Table 3:** Voxel-based morphometry results

Cluster level statistics				MNI coordinates (mm)		
*P*_uncorr_	*P*_FWE-corr_	*q*_FDR-corr_	*k*_*E*_	*x*	*y*	*z*
< 0.0001	< 0.0001	< 0.0001	855	36	2	− 6
< 0.0001	< 0.0001	< 0.0001	1669	4	− 74	20
< 0.0001	< 0.0001	< 0.0001	247	42	− 22	20
< 0.0001	< 0.0001	< 0.0001	2098	− 50	− 30	8
< 0.0001	< 0.0001	< 0.0001	82	54	− 2	10
< 0.0001	< 0.0001	< 0.0001	199	42	− 64	4
< 0.0001	< 0.0001	< 0.0001	750	60	− 40	2
< 0.0001	< 0.0001	< 0.0001	286	− 6	32	− 14
< 0.0001	< 0.0001	< 0.0001	77	6	36	14
< 0.0001	< 0.0001	< 0.0001	39	28	− 34	− 6
< 0.0001	< 0.0001	< 0.0001	23	− 30	10	8
< 0.0001	< 0.0001	< 0.0001	90	− 58	− 2	26

Group-level voxel-wise statistical parametric map derived from VBM analysis showing sCVR reductions in older adults compared to younger adults. Twelve statically significant clusters were identified centered around regions including left medial frontal cortex, bilateral anterior cingulate gyrus, bilateral anterior insula, bilateral posterior insula, bilateral parietal and central operculum, bilateral precuneus, right calcarine cortex, right cuneus, right inferior and middle occipital gyrus, bilateral transverse temporal gyrus, left superior temporal gyrus, bilateral planum polare, right hippocampus, right putamen, and right thalamus.

Cluster level statistics with uncorrected *p* value, family-wise error (FWE) *p* value, false discovery rate (FDR) corrected *q* value, and cluster size in voxels (*k*_*E*_) displayed alongside a *t*-statistic color bar with higher *t*-statistic indicating greater differences in sCVR in older adults compared to younger adults, with lighter colors indicating great reduction. Montreal Neurological Institute** (**MNI) coordinates along the *y*-axis indicated for each axial slice.

### Sensitivity analysis

Sensitivity analyses were performed utilizing a smaller cohort of older adults, specifically.

older adults with low VRF burden. Inferential statistics revealed similar results as the broader analysis, identifying both whole brain and regional reductions in sCVR in older adults compared to younger adults. Notably, while many regions exhibited attenuated, sCVR in older adults, effects sizes were largest in medial temporal regions including the entorhinal cortex and the parahippocampal gyrus (Supplementary Table 2). An additional sensitivity analysis was conducted using a smaller cohort of older adults matched by sex and sample size to the younger adult cohort in order to determine the potential effect of unbalanced sample size (Supplemental Table 3). Inferential statistics revealed results consistent with the main analysis, identifying both whole brain and regional deficits in sCVR in older adults compared to younger adults. Large effect sizes were seen across nearly all regions of the brain, with the largest in the precuneus and amygdala. Lastly, in order to account for the potential influence of blood pressure on sCVR, a sensitivity analyses was done controlling for MAP, VRF burden, and sex in a subset of participants in which MAP was available (Supplementary Table 4). Inferential statistics revealed results consistent with the main analysis. While some regions attenuated, whole brain, medial temporal regions, and the precuneus remained significant with large effect sizes.

## Discussion

The present study is the first to investigate age group differences in CVR by directly comparing sCVR responses at rest in younger and older adults. This study found attenuated sCVR at rest in cognitively unimpaired older adults relative to healthy younger adults in the whole brain and in nearly all regions examined, independent of mean CBF. These findings are consistent with previously reported deficits in CVR responses to experimentally induced hyper- and hypocapnic conditions in older adults relative to younger adults[9, 20–25]. Importantly, these results highlight that age-related deficits in cerebrovascular function are apparent in spontaneous fluctuations in blood flow and CO_2_ at rest, underscoring their potential importance in brain health under naturalistic conditions. Prior studies from our group and others have suggested that both sCVR and experimentally induced CVR responses are deficient in older adults with vascular brain injury and cognitive impairment relative to older adults with minimal cerebrovascular disease and no cognitive impairment[4, 8, 10]. In the present study, we observed deficits in sCVR in cognitively unimpaired older adults relative to younger adults, a finding which remained significant in an older adult subsample with low vascular risk (0 to 1 risk factor). These findings suggest sCVR at rest may offer potential utility as an early indicator of vascular dysfunction in older adults that may predate irreversible vascular brain injury and cognitive impairment. Further longitudinal research is needed to determine whether deficits in sCVR at rest presage future vascular brain injury and cognitive decline.

Results from ROI analyses highlight widespread age-group differences in sCVR at rest in essentially all cortical, subcortical, and limbic regions examined. This is consistent with prior studies on age group differences in experimentally induced CVR which has also found widespread differences [23], while results from other studies have suggested that frontal and temporal regions may be the earliest to show decline in CVR responses [9, 20]. The largest effect sizes observed in the present study were within the medial temporal lobe in both primary and sensitivity analyses, which is consistent with findings from our prior study of age differences in experimentally induced CVR[23]. We also observed deficits in sCVR and experimentally induced CVR responses in medial temporal regions in older adults with cognitive impairment compared to those who were cognitively unimpaired [8]. Another recent study also found a decrease in hippocampal pericytes in individuals with vascular dementia, Alzheimer’s disease, and post-stroke [43]. Taken together with the results of the present study, these findings could suggest age-related vulnerability of medial temporal regions to cerebrovascular dysfunction, with major implications for development of memory impairment in older adults.

Here we also report that cognitively unimpaired older adults show decreased whole brain CBF and CBF variability at rest compared to younger adults. Differences in CBF variability between groups were also significant in models controlling for mean CBF, suggesting that in older adults the reduced spontaneous fluctuations in CBF at rest are independent of differences in resting mean CBF alone. This suggests that even in relatively healthy older adults with low vascular risk and no cognitive impairment, reductions in both the baseline CBF and the natural fluctuations of CBF are observed, potentially indicating early microvascular changes that may contribute to age-related risk for vascular brain injury [2, 6]. Notably, age group differences in CBF variability and sCVR were more substantial than that of mean CBF, suggesting that CBF variability and sCVR may be more sensitive measures of age-related cerebrovascular functional changes.

Notably, based on the voxel-wise analysis, we observed more pronounced reductions in sCVR in the right hemisphere compared to the left. This finding aligns with prior literature suggesting greater vulnerability of the right hemisphere to age-related structural and functional decline[44, 45]. These lateralized effects may suggest asymmetries in vascular or metabolic aging and highlight the importance of considering hemispheric differences when interpreting age-related cerebrovascular changes. Additionally, in our older adult cohort, handedness data indicated that the majority were right-handed (see Table 1). Handedness data were not available for the younger adults. Given the predominance of right-handed individuals, the observed right-hemisphere reductions in sCVR are unlikely to be explained solely by handedness and instead may reflect a broader right-lateralized vulnerability in cerebrovascular aging, consistent with prior reports[44, 45]. However, little is currently known about the lateralization of cerebrovascular reactivity itself or broader patterns of vascular asymmetry in aging. Future research should investigate laterality of CVR responses, how they change with age, and whether they contribute meaningfully to age-related brain vulnerability or resilience.

Additionally, taken together with the recent findings implicating deficits in sCVR at rest with cognitive impairment[8], results highlight the potential utility of measuring sCVR at rest is in part due to its relative simplicity and tolerability when compared with other methods utilizing manipulation of blood and CSF CO_2_ levels through gas administration or breathing paradigms. A key strength of the present study it the computation of sCVR using _ET_CO_2._ Further research is necessary to compare methods of measuring and quantifying CVR in order to better understand the relative advantages and disadvantages of various approaches to sCVR at rest.

Some limitations of this study include the cross-sectional design and relative lack of diversity in the sample. Additionally, while a strength of the present study was its comparison of sCVR in two distinct age groups, the sample size of the younger adult cohort was relatively low, and these groups do not encompass the entire lifespan; thus, findings may not capture differences in CVR responses in other age groups or across the lifespan, as some literature suggests that decline in CVR responses begin and is most rapid during the middle age [9]. Future longitudinal research on sCVR at rest is warranted to better understand how changes occur across the lifespan. The present study also only evaluated CBF and sCVR in the grey matter only as the current sequence was not optimized to capture white matter CBF. Although the use of pCASL MRI is a strength as it is a direct quantitative measure of perfusion in the brain, we were not able to temporally shift _ET_CO_2_ data or adjust for blood transit time from the lung to the brain in our method of processing _ET_CO_2_ due to the low temporal resolution of the pCASL images. Lastly, although sCVR responses are primarily interpreted as reflecting fluctuations in end-tidal CO₂, it is important to note that blood pressure variability and spontaneous neural activity (via neurovascular coupling) also contribute to resting-state CBF changes, and thus sCVR likely reflects the combined influence of these factors. Future research should incorporate simultaneous measures of end-tidal CO₂, arterial blood pressure, and neural activity to disentangle their relative contributions to sCVR and better characterize the interplay of vascular and neural mechanisms underlying resting-state CBF fluctuations.

## Conclusion

Our findings indicate that relative to younger adults, cognitively unimpaired older adults exhibit deficits in sCVR at rest. These deficits were found in whole brain sCVR responses and regional sCVR in nearly all regions examined. Compared to younger adults, older adults exhibited lower resting whole brain CBF and CBF variability. These results suggest that aging may have a disproportionate impact on the ability of the vasculature to respond to physiologic demand, even in the absence of hypo- or hypercapnic challenge. The ability of sCVR responses to capture microvascular changes associated with aging may be of great value due to the relative simplicity and tolerability of this resting state approach relative to administration of gas or guided breathing. Taken together, these results highlight that a resting approach to sCVR responses at rest may be indicative of early microvascular dysfunction in aging individuals while also suggesting that sCVR changes occur as individuals age, even in the absence of concurrent vascular risk or cognitive decline.

## Supplementary Information

Below is the link to the electronic supplementary material.

ESM 1 (DOCX 28.0 KB)

ESM 2 (DOCX 20.9 KB)

ESM 3 (DOCX 21.0 KB)

ESM 4 (DOCX 28.1 KB)

## Data Availability

All data analyzed in the study are available from the corresponding author on reasonable request.

## Abbreviations

BBB: Blood-brain barrier
CBF: Cerebral blood flow
CVR: Cerebrovascular reactivity
_ET_CO2: End-tidal carbon dioxide
pCASL: Pseudo-continuous arterial spin labeling
TCD: Transcranial doppler
VBM: Voxel-based morphometry
