# Supplementary material for: Age differences in spontaneous cerebrovascular reactivity at rest
Source: GeroScience. 2025 Oct 30;48(4):5547–60. doi: 10.1007/s11357-025-01935-9 (PMC13575053; doi:10.1007/s11357-025-01935-9)
Supplement: Supplementary file 1 [file 11357_2025_1935_MOESM1_ESM.docx]

| **Supplemental Table 1:** Comparison of mean regional spontaneous CVR values for younger and older adults, sex-matched and controlling for mean CBF and sex | | | | | | | |
| --- | --- | --- | --- | --- | --- | --- | --- |
| **Brain Region** | **Younger**  **Adults**  **(N = 24)**  *M (SD)* | **Older**  **Adults**  **(N = 129)**  *M (SD)* | **Mean Difference**  *M (SE)* | **F** | ***p*** | **ηp^2^** |  |
| Whole Brain | 45.83 (33.61) | 21.06 (11.08) | 24.96 (3.73) | 17.20 | <0.001 | 0.104 |  |
| Superior Frontal Gyrus | 28.89 (16.93) | 20.35 (14.18) | 8.55 (3.27) | 2.56 | 0.112 | 0.020 |  |
| Middle Frontal Gyrus | 35.45 (31.33) | 21.51 (14.63) | 14.13 (4.11) | 6.44 | 0.012 | 0.045 |  |
| Inferior Frontal Gyrus | 53.54 (44.02) | 27.27 (17.61) | 25.96 (5.01) | 8.64 | 0.004 | 0.055 |  |
| Medial Orbitofrontal Cortex | 61.45 (49.78) | 32.34 (26.75) | 30.26 (7.86) | 8.27 | 0.005 | 0.062 |  |
| Lateral Orbitofrontal Cortex | 57.80 (49.51) | 32.19 (24.97) | 25.91 (7.09) | 4.06 | 0.046 | 0.029 |  |
| Anterior Cingulate Cortex | 44.37 (29.59) | 27.27 (16.94) | 17.85 (4.49) | 4.93 | 0.028 | 0.035 |  |
| Posterior Cingulate Cortex | 29.40 (17.39) | 19.61 (12.95) | 12.01 (3.18) | 7.98 | 0.005 | 0.057 |  |
| Precuneus | 36.75 (21.91) | 21.12 (13.43) | 16.35 (3.42) | 8.15 | 0.005 | 0.053 |  |
| Inferior Parietal Cortex | 34.79 (27.51) | 19.08 (16.39) | 15.46 (4.39) | 7.44 | 0.007 | 0.053 |  |
| Inferior Temporal Gyrus | 35.34 (26.87) | 19.57 (13.68) | 15.85 (3.99) | 6.77 | 0.010 | 0.049 |  |
| Medial Temporal Lobe | 47.88 (36.02) | 18.78 (14.14) | 29.12 (4.52) | 20.46 | <0.001 | 0.132 |  |
| Perirhinal Cortex | 33.94 (22.24) | 24.96 (18.45) | 9.66 (4.54) | 0.28 | 0.284 | 0.002 |  |
| Entorhinal Cortex | 46.91 (31.93) | 21.03 (15.05) | 26.70 (4.67) | 18.40 | <0.001 | 0.139 |  |
| Parahippocampal Gyrus | 51.01 (36.78) | 20.45 (13.87) | 30.33 (4.49) | 24.32 | <0.001 | 0.149* |  |
| Hippocampus | 42.13 (32.99) | 22.41 (15.15) | 19.82 (4.50) | 9.68 | 0.002 | 0.067 |  |
| Amygdala | 53.87 (34.77) | 29.01 (19.53) | 24.87 (5.24) | 6.73 | 0.011 | 0.049 |  |
| Caudate | 45.14 (38.17) | 24.15 (17.55) | 31.49 (5.31) | 4.420 | 0.038 | 0.035 |  |
| Thalamus | 30.10 (19.72) | 21.84 (17.07) | 7.74 (4,13) | 1.38 | 0.242 | 0.011 |  |

* Large effect sizes: ηp^2^>0.14 (Cohen, 1988)
