# Supplementary material for: Age differences in spontaneous cerebrovascular reactivity at rest
Source: GeroScience. 2025 Oct 30;48(4):5547–60. doi: 10.1007/s11357-025-01935-9 (PMC13575053; doi:10.1007/s11357-025-01935-9)
Supplement: Supplementary file 2 [file 11357_2025_1935_MOESM2_ESM.docx]

| **Supplemental Table 2:** Comparison of mean regional spontaneous CVR values for younger and older adults with low VRF burden at rest, controlling for sex | | | | | | | |  |
| --- | --- | --- | --- | --- | --- | --- | --- | --- |
| **Brain Region** | **Younger**  **Adults**  **(N = 24)**  *M (SD)* | **Older**  **Adults**  **(N = 87)**  *M (SD)* | **Mean Difference**  *M (SE)* | **F** | ***p*** | **η_p_^2^** | | |
| Whole Brain | 45.83 (33.61) | 20.73 (11.74) | 25.09 (4.34) | 33.45 | <0.001 | 0.236 | | |
| Superior Frontal Gyrus | 28.89 (16.93) | 20.08 (12.27) | 8.98 (3.30) | 7.40 | 0.008 | 0.074 | | |
| Middle Frontal Gyrus | 35.45 (31.33) | 20.93 (14.51) | 14.67 (4.57) | 10.30 | 0.002 | 0.093 | | |
| Inferior Frontal Gyrus | 53.5 (44.02) | 26.85 (17.54) | 26.08 (6.08) | 18.39 | <0.001 | 0.150* | | |
| Medial Orbitofrontal Cortex | 61.45 (49.78) | 37.92 (25.89) | 26.96 (8.41) | 10.27 | 0.002 | 0.102 | | |
| Lateral Orbitofrontal Cortex | 57.80 (49.51) | 33.23 (26.02) | 24.71 (7.86) | 9.87 | 0.002 | 0.088 | | |
| Anterior Cingulate Cortex | 44.37 (29.57) | 30.47 (17.11) | 16.83 (4.91) | 11.75 | <0.001 | 0.105 | | |
| Posterior Cingulate Cortex | 29.40 (17.39) | 16.95 (12.60) | 12.34 (3.21) | 14.74 | <0.001 | 0.131 | | |
| Precuneus | 36.75 (21.91) | 20.19 (12.87) | 16.96 (3.59) | 22.33 | <0.001 | 0.171* | | |
| Inferior Parietal Cortex | 34.79 (27.51) | 19.08 (16.60) | 16.40 (4.75) | 11.95 | <0.001 | 0.109 | | |
| Inferior Temporal Gyrus | 35.34 (26.87) | 19.13 (13.56) | 16.26 (4.34) | 14.01 | <0.001 | 0.130 | | |
| Medial Temporal Lobe | 47.88 (36.02) | 18.74 (14.34) | 29.41 (5.04) | 34.02 | <0.001 | 0.252* | | |
| Perirhinal Cortex | 33.94 (22.24) | 26.83 (19.99) | 7.53 (5.06) | 2.21 | 0.140 | 0.022 | | |
| Entorhinal Cortex | 46.91 (31.93) | 20.03 (14.24) | 27.59 (4.99) | 30.45 | <0.001 | 0.261* | | |
| Parahippocampal Gyrus | 51.01 (36.78) | 19.65 (13.62) | 31.48 (4.99) | 39.84 | <0.001 | 0.279* | | |
| Hippocampus | 42.13 (32.99) | 22.09 (15.38) | 20.15 (4.95) | 16.61 | <0.001 | 0.141* | | |
| Amygdala | 53.87 (34.77) | 27.75 (19.83) | 26.14 (5.66) | 21.34 | <0.001 | 0.173* | | |
| Caudate | 45.14 (38.17) | 25.78 (19.71) | 19.53 (5.38) | 10.18 | 0.002 | 0.098 | | |
| Thalamus | 30.10 (19.72) | 22.58 (15.78) | 7.38 (4.12) | 3.22 | 0.076 | 0.034 |  |  |

* Large effect sizes: η_p_^2^>0.14 (Cohen, 1988)
