# Supplementary material for: Age differences in spontaneous cerebrovascular reactivity at rest
Source: GeroScience. 2025 Oct 30;48(4):5547–60. doi: 10.1007/s11357-025-01935-9 (PMC13575053; doi:10.1007/s11357-025-01935-9)
Supplement: Supplementary file 3 [file 11357_2025_1935_MOESM3_ESM.docx]

| **Supplemental Table 3:** Comparison of mean regional spontaneous CVR values for younger and older adults, sample size matched, sex-matched and controlling for sex | | | | | | | |  |
| --- | --- | --- | --- | --- | --- | --- | --- | --- |
| **Brain Region** | **Younger**  **Adults**  **(N = 24)**  *M (SD)* | **Older**  **Adults**  **(N = 24)**  *M (SD)* | **Mean Difference**  *M (SE)* | **F** | ***p*** | **η_p_^2^** | | |
| Whole Brain | 45.83 (33.61) | 15.02 (5.27) | 30.80 (7.02) | 19.27 | <0.001 | 0.300 | | |
| Superior Frontal Gyrus | 28.89 (16.93) | 17.11 (14.97) | 11.75 (4.80) | 5.99 | 0.019 | 0.125 | | |
| Middle Frontal Gyrus | 35.45 (31.33) | 16.68 (11.26) | 18.77 (7.00) | 8.05 | 0.007 | 0.158* | | |
| Inferior Frontal Gyrus | 53.54 (44.02) | 20.74 (14.43) | 32.72 (9.60) | 11.61 | 0.001 | 0.213* | | |
| Medial Orbitofrontal Cortex | 61.45 (49.78) | 19.68 (12.22) | 41.77 (11.65) | 12.86 | 0.001 | 0.269* | | |
| Lateral Orbitofrontal Cortex | 57.80 (49.51) | 17.29 (13.10) | 40.19 (11.68) | 11.84 | 0.001 | 0.238* | | |
| Anterior Cingulate Cortex | 44.37 (29.57) | 19.63 (11.87) | 24.74 (6.63) | 14.30 | <0.001 | 0.259* | | |
| Posterior Cingulate Cortex | 29.40 (17.39) | 14.68 (11.65 | 14.72 (4.39) | 13.26 | <0.001 | 0.244* | | |
| Precuneus | 36.75 (21.91) | 15.29 (8.99) | 21.60 (4.89) | 19.49 | <0.001 | 0.307* | | |
| Inferior Parietal Cortex | 34.79 (27.51) | 17.21 (16.24) | 17.61 (7.01) | 6.31 | 0.016 | 0.136 | | |
| Inferior Temporal Gyrus | 35.34 (26.87) | 16.24 (9.39) | 19.10 (6.45) | 8.77 | 0.005 | 0.192* | | |
| Medial Temporal Lobe | 47.88 (36.02) | 16.47 (13.17) | 31.51 (8.29) | 14.45 | <0.001 | 0.261* | | |
| Perirhinal Cortex | 33.94 (22.24) | 19.03 (11.12) | 15.17 (5.34) | 8.07 | 0.007 | 0.168* | | |
| Entorhinal Cortex | 46.91 (31.93) | 19.40 (17.83) | 28.01 (8.64) | 10.52 | 0.003 | 0.231* | | |
| Parahippocampal Gyrus | 51.01 (36.78) | 20.69 (15.02) | 30.21 (8.77) | 11.86 | 0.001 | 0.229* | | |
| Hippocampus | 42.13 (32.99) | 20.54 (14.43) | 23.71 (7.73) | 9.40 | 0.004 | 0.194* | | |
| Amygdala | 53.87 (34.77) | 21.12 (17.27) | 32.75 (8.47) | 17.24 | <0.001 | 0.307* | | |
| Caudate | 45.14 (38.17) | 18.75 (11.95) | 19.53 (5.38) | 7.82 | 0.008 | 0.174* | | |
| Thalamus | 30.10 (19.72) | 18.69 (12.78) | 11.41 (5.08) | 6.29 | 0.016 | 0.139 |  |  |

* Large effect sizes: η_p_^2^>0.14 (Cohen, 1988)
