# Supplementary material for: Age differences in spontaneous cerebrovascular reactivity at rest
Source: GeroScience. 2025 Oct 30;48(4):5547–60. doi: 10.1007/s11357-025-01935-9 (PMC13575053; doi:10.1007/s11357-025-01935-9)
Supplement: Supplementary file 4 [file 11357_2025_1935_MOESM4_ESM.docx]

| **Supplementary Table 4:** Comparison of mean regional spontaneous CVR values for younger and older adults at rest, controlling for sex, VRF burden, and MAP. | | | | | | |
| --- | --- | --- | --- | --- | --- | --- |
| **Brain Region** | **Younger**  **Adults**  **(N = 18)**  *M (SD)* | **Older**  **Adults**  **(N = 107)**  *M (SD)* | **Mean Difference**  *M (SE)* | **F** | ***p*** | **η_p_^2^** |
| Whole Brain | 41.95 (28.63) | 21.24 (10.78) | 20.27 (4.12) | 24.25 | <0.001 | 0.185* |
| Superior Frontal Gyrus | 29.22(17.31) | 19.85 (12.98) | 9.08 (4.00) | 5.160 | 0.025 | 0.048 |
| Middle Frontal Gyrus | 32.16 (27.97) | 20.81 (13.86) | 11.71 (4.73) | 6.130 | 0.015 | 0.051 |
| Inferior Frontal Gyrus | 53.68 (42.93) | 28.02 (17.23) | 25.36 (6.41) | 25.66 | <0.001 | 0.115 |
| Medial Orbitofrontal Cortex | 56.29 (48.42) | 33.08 (26.78) | 21.66 (9.65) | 5.04 | 0.027 | 0.047 |
| Lateral Orbitofrontal Cortex | 58.91 (49.25) | 34.38 (25.75) | 29.00 (8.63) | 11.28 | 0.001 | 0.089 |
| Anterior Cingulate Cortex | 46.39 (29.43) | 27.21 (16.67) | 17.54 (5.23) | 10.43 | 0.002 | 0.0.85 |
| Posterior Cingulate Cortex | 30.07 (17.67) | 17.59 (12.98) | 14.29 (3.91) | 13.34 | <0.001 | 0.106 |
| Precuneus | 37.41 (22.39) | 21.04(13.20) | 18.77 (4.17) | 20.23 | <0.001 | 0.143* |
| Inferior Parietal Cortex | 31.86 (21.36) | 20.95 (16.90) | 15.86 (4.91) | 10.43 | 0.002 | 0.089 |
| Inferior Temporal Gyrus | 35.67 (28.77) | 19.54 (13.11) | 16.10 (4.85) | 11.01 | 0.001 | 0.091 |
| Medial Temporal Lobe | 44.95 (37.17) | 18.77 (14.99) | 29.06 (5.77) | 25.36 | <0.001 | 0.185* |
| Perirhinal Cortex | 31.14 (21.65) | 25.10 (18.08) | 3.01 (5.39) | 0.31 | 0.578 | 0.003 |
| Entorhinal Cortex | 44.09 (33.30) | 18.85 (14.65) | 27.85 (5.85) | 22.70 | <0.001 | 0.196* |
| Parahippocampal Gyrus | 47.45 (37.92) | 19.91 (14.03) | 28.38 (5.54) | 26.27 | <0.001 | 0.187* |
| Hippocampus | 39.45 (33.08) | 21.53 (15.28) | 18.81 (5.47) | 11.84 | <0.001 | 0.095 |
| Amygdala | 49.85 (34.24) | 30.16 (20.23) | 24.27 (6.60) | 13.50 | <0.001 | 0.108 |
| Caudate | 42.82 (36.79) | 23.48 (17.15) | 17.37 (6.29) | 16.39 | 0.007 | 0.071 |
| Thalamus | 29.32 (20.00) | 21.71 (17.03) | 6.83 (5.12) | 1.78 | 0.185 | 0.016 |

* Large effect sizes: η_p_^2^>0.14 [42]
